# Longitudinal study of the effects of price and promotion incentives on purchases of unhealthy foods: evidence for restricting food promotions

**DOI:** 10.1136/bmjnph-2021-000323

**Published:** 2022-03-04

**Authors:** Daniel Kopasker, Ourega-Zoé Ejebu, Patricia Norwood, Anne Ludbrook

**Affiliations:** 1 MRC/CSO Social and Public Health Sciences Unit, University of Glasgow, Glasgow, UK; 2 Health Economics Research Unit, University of Aberdeen, Aberdeen, UK; 3 School of Health Sciences, University of Southampton, Southampton, Hampshire, UK; 4 Rowett Institute, University of Aberdeen, Aberdeen, UK

**Keywords:** dietary patterns

## Abstract

**Objectives:**

Taxes and restrictions on promotions have recently been proposed as policy instruments to reduce consumption of unhealthy foods. The objective of this study is to add to the limited evidence on the comparative effectiveness of price changes, price promotions and volume promotions in changing household purchasing of unhealthy foods, using biscuits, crisps and savoury snacks as examples.

**Design:**

Longitudinal regression analysis of consumer microdata.

**Setting:**

Secondary data on itemised household purchases of biscuits, crisps and savoury snacks from 2006 to 2012.

**Participants:**

Sample of 3024 households in Scotland.

**Main outcome measures:**

Changes in the number of calories (kcal) purchased in the product category by a household caused by changes in the price for the product category, any temporary in-store price promotions and any temporary in-store volume promotions. Changes are measured at the mean, median, 25th percentile and 75th percentile of the household purchasing distribution for the full sample. Subgroup analyses were conducted by household income band and for households with and without children.

**Results:**

Between product categories, the scale of purchasing response to incentives varies significantly. Within product categories, the mean calories (kcal) purchased by a household are more responsive to any volume promotion than to price or any price promotion for all product categories. As the volume of items purchased increases, households are less responsive to price, less responsive to any volume promotion and more responsive to any price promotion. Statistically significant differences are observed between household income groups in their response to price and promotion incentives within the biscuits category only. In cases where statistically significant differences are observed, households with children are more responsive to promotion and price incentives than households without children.

**Conclusions:**

For all product categories analysed (biscuits, crisps and savoury snacks), household purchasing is most responsive to any volume promotion. Therefore, assuming the response of consumers to incentives remains constant following legislation, the most effective policy instrument to reduce the calorie intake from these products may be a ban on volume promotions.

**Key messages**

**What is already known on this topic**

- Foods high in calories, fat, sugar or salt (HFSS) are purchased on promotion more often than healthier products.
- Policies based on price interventions can be effective in reducing consumption of unhealthy products.

**What this study adds**

- This paper estimates the separate effects of price, price promotions and volume promotions on purchases of HFSS products.

**How this study might affect research, practice or policy**

- The analysis clearly indicates the most effective target for a policy instrument aiming to reduce purchases of HFSS products.

## Introduction

Tackling obesity and promoting healthy diets have been longstanding policy objectives for many governments and international bodies such as the European Commission and WHO.^1 2^ However, progress towards meeting these objectives has been limited. For example, in 1996 Eating for Health: A Diet Plan for Scotland was published,^3^ yet over 20 years later many of the same issues were discussed in A Healthier Future—Scotland’s Diet and Healthy Weight Delivery Plan.^4^ The Restricting Food Promotions Bill was expected to be included in the legislative programme of the Scottish Government for the 2020/2021 session but was subsequently delayed while the implications for the bill of the coronavirus pandemic are considered.^5 6^ Conversely, the link between obesity and COVID-19 outcomes was stated as one motivation for the UK Government’s decision to legislate for England to restrict the locations and promotion of energy dense foods high in calories, fat, sugar or salt (HFSS).^7^ Discretionary foods, which are the focus of the proposed Scottish legislation, are a subset of HFSS foods which have no nutritional benefit other than energy, with purchases accounting for almost one-fifth (19%) of total calories, total fat and saturated fats in the Scottish diet.^8 9^ The product categories analysed in this paper meet the definitions of discretionary and HFSS foods and are referred to as HFSS foods hereafter.

One way to improve the quality of diets and address obesity is to inform the design of policy instruments by observing and understanding household purchasing behaviour for HFSS foods. Price interventions to reduce consumption of unhealthy products have been successfully applied in other areas, such as tobacco and alcohol consumption, but have had less use in relation to healthy diets.^10^ The recent introduction of the Soft Drinks Industry Levy by the UK Government is an example of successful innovation in this policy area, achieving a greater reduction in sugar content than achieved through voluntary agreements with the food industry.^11^ The levy resulted in reductions in sugar content to avoid the levy and large price increases for products which were not reformulated.^12^

Restrictions on the promotion of HFSS products, the focus of legislation currently being proposed by the Scottish and UK governments, is a further potential policy instrument. Restrictions on promotions may be particularly effective since HFSS foods tend to be purchased more frequently on promotion than healthier products.^8 9^ A systematic review also found that,^13^ in the majority of studies, promotions were more likely on unhealthy foods and accounted for a greater share of promotions. Furthermore, there is evidence that promotions contribute to consumers purchasing more than expected, both within the promoted product category and overall.^14^ Analysis of sales by Nakamura *et al* found no significant difference in promotions being applied to healthy and unhealthy products but did find that the effect on sales was greater for unhealthy foods.^15^

While a number of studies have analysed the effect of any type of promotion, including examples for the UK,^15 16^ this paper makes a distinct contribution by considering the separate effects of price, price promotions and volume promotions. Detailed consumer panel data are used to predict the potential reductions in household purchasing of three categories of HFSS products achievable through three distinct policy instruments: a policy instrument such as a tax that directly changes the price of a product, restrictions on promotions which temporarily reduce the price of a product at a given volume, and restrictions on promotions that increase the volume of a product at a given price. Since the primary motivation for a policy response is to address obesity, particularly child obesity, we investigate variation in demand responsiveness to in-store incentives across the household demand distribution, and between households with and without children. Respondents to a public consultation on the UK Government’s planned legislation highlighted concerns that policy instruments may disproportionately affect households in lower socioeconomic groups.^7^ To address this concern, we additionally investigate potential heterogeneity in demand responsiveness across the household income distribution. Demand responsiveness within specific consumer strata, such as high-volume consumers or households with children, is also investigated. By analysing observed changes in demand resulting from different in-store incentives, and making assumptions regarding how demand would change if the incentives were removed, it is possible to inform the choice of an effective target for policy instrument aiming to reduce consumption in distinct categories of HFSS products.

## Methods

Regression models are estimated using household expenditure data from the Kantar Worldpanel (KWP). This secondary dataset consists of detailed microdata on individual food purchases made by households for home consumption from a range of outlets. Product information is recorded by participants after a purchase using in-home handheld barcode scanners. This process has the advantage of providing accurate product-level information on characteristics including price, quantity, energy content and details of any promotions. Nutritional information is updated annually by KWP using food package labels. Information for new products is added throughout the year. KWP periodically (in this instance every 5 years) collects sociodemographic information on households. The date at which a household joined the KWP is also provided. Most of the sociodemographic characteristics included in the model have been adjusted to reliably reflect time points between data collection in 2007 and 2012. Where this was not possible, variables are fixed between data collection points.

The responsiveness of consumers to price, price promotions and volume promotions is estimated through a series of regression models using longitudinal household purchasing data for three distinct product categories (biscuits, crisps and savoury snacks) which meet the definition of HFSS foods. The product categories are formed by aggregating product-level data. These categories are selected as they are among the most common HFSS foods purchased by households, are included within the HFSS product categories in the UK government’s proposed legislation,^7^ and have reliable nutritional information available. As far as possible, the products included within each product category are based on those outlined in the UK government’s public consultation on HFSS legislation.^7^ Biscuits are defined as all types of sweet biscuits, including cereal bars, and individually wrapped single-serve chocolate biscuit bars. Crisps are defined as all standard potato, grain, vegetable and pulse-based crisps. Savoury snacks include items such as popcorn, pretzels and poppadums. Since the focus of the analysis is identifying policy instruments to reduce obesity, demand for a product category is measured in calories (kcal) purchased. Only non-zero purchases are observed due to the data collection process. We have not attempted to address this limitation of the data as imputation of price and promotion characteristics for zero purchases would be highly unreliable and these variables are essential to the focus of the analysis. Also, there is no scope to reduce zero purchases, so such instances have little relevance to analysis concerning ways to reduce the volume of purchases.

Analysis is conducted on a sample of Scottish households (n=3024) that made at least one purchase of biscuits, crisps and savoury snacks over the sample period (2006–2012). Prices were adjusted for inflation using the ONS annual index D7BT with 2010 as the base year. Product-level data from each day is aggregated to weekly category-level data for analysis. This results in 362 time periods. The sample is additionally restricted to households with no missing data on household income. Four hundred fifty-five households are not included within the sample due to missing household income information. As heterogeneity in demand responsiveness between household income subgroups is of policy relevance, this restriction was necessary. However, sensitivity analysis was conducted to test the influence of sample restrictions on the outcomes of interest.

Each model estimates the constant own-price elasticity of demand (the change in demand for a product for a given change in price), while simultaneously estimating the percentage change in demand on occasions when the relevant product category is being promoted in distinct forms (price or volume), holding all else constant. In models of aggregate demand, it has been suggested that price and volume are endogenous.^17^ However, as our study concentrates on individual household demand, the scope for households to influence the market price within product categories is very limited.^18^

The promotions variables included in the model do not disaggregate to specific price or volume promotions, they indicate that at least one purchase was made on either any price promotion or any volume promotion in a week. The model allows for households to have made purchases on both price and volume promotions in a given week. The price of a product is expressed in terms of pence per gram. Volume promotions affect the number of grams, and price promotions affect the number of pence. Therefore, the price effect of a promotion is captured by the price coefficient. The promotion coefficients capture the effect of a promotion over and above the price effect of the promotion. When reporting the price coefficient, a 10% price change is used in the main text to aid presentation by enhancing comparability with the promotions coefficients. The model was estimated based on a 1% change in price and these results are reported in the online supplemental appendix.

10.1136/bmjnph-2021-000323.supp1 Supplementary data

The model specification to estimate the effects of price or promotions on demand takes the form:

$$LogD_{cht}=\beta_{1}LogP_{cht}+\beta_{2}X_{cht}+\beta_{3}V_{cht}+{Z^{\prime }}_{ht}\gamma +\alpha_{h}+\eta_{y}+\varepsilon_{ht}$$

where *D* is the calories (kcal) purchased of product category *c* by household *h* at time *t*. The log transformation does not result in a loss of data since only non-zero purchases are within the dataset. *P* is the real price per gram paid by the household, *X* is a dummy variable indicating that the household made at least one purchase during a price promotion in the week, *V* is an equivalent dummy variable for volume promotions, *Z* is a vector of household characteristics (age of main shopper, age of main shopper squared divided by 100, household size, time since last purchase of product category and time in panel). Unobserved household heterogeneity is captured by the household fixed effect *α. η* is a dummy variable for year *y* (base year is 2006), and *ε* is an idiosyncratic error term. SEs are clustered at the household level such that they are robust to arbitrary heteroscedasticity and within-household autocorrelation. For quantile regressions, clustered SEs are obtained by bootstrapping.

The age of the main shopper (and its square) is included within the model to control for variation in purchasing at different life stages. Household size controls for larger households having additional energy wants and needs. Time since the last purchase controls for potential stockholding by households. The time a household has been in the panel controls for possible measurement error due to respondent fatigue, a potential issue in consumer panel data.^19^ There is substantial variation in time spent in the panel, with the mean level for the sample being 1.7 years but ranging from new entrants to almost 19 years.

Where any of the household characteristics are time-invariant over the sample period, the household fixed effect will control for these features. As such, the preceding variables control for only the time-varying element of the household characteristics.

The base model estimates the effects of price and promotions on demand at the mean. To move beyond the mean and estimate effects among high-volume and low-volume consumers, we use the panel quantile regression methods to estimate the effect on demand at the 25th, 50th and 75th percentiles.^20^ Subgroup analyses (at the mean) are conducted based on household income and having children in the household. A series of χ^2^ tests are used to investigate differences in response to price and promotion both within and between product categories.

Patients or the public were not involved in the design, or conduct, or reporting, or dissemination plans of the research.

## Results

Table 1 provides the level of calories (kcal) purchased per household member at key points within the household purchasing distribution. The sample of households is constant across product categories, but the number of observations varies between categories since the frequency of purchasing differs between households. At the mean, over 500 calories (kcal) are purchased per person per week for all product categories analysed. There is substantial variation in the level of calories (kcal) purchased both within and between product categories. Almost twice as many calories (kcal) are purchased in biscuits compared with savoury snacks. Within category, approximately 70% more calories (kcal) per person are purchased at the 75th percentile compared with the median for biscuits and savoury snacks. The equivalent figure for crisps is closer to 60%. As expected, a substantial proportion of HFSS purchases are made during promotions. Across all product categories, approximately 40% of calories (kcal) are purchased in a week when at least one purchase was made on either a price or volume promotion. This rate is comparable with those observed within higher sugar product categories reported by Public Health England.^14^ For biscuits only, more calories per person in the household are purchased on price promotion than on volume promotion.

**Table 1:** Descriptive statistics for household purchasing of biscuits, crisps and savoury snacks

	Biscuits	Crisps	Savoury snacks
Weekly calories (kcal) purchased per person in household			
Mean	1239	801	665
25th percentile	493	377	287
Median	917	624	489
75th percentile	1563	999	848
Mean percentage of calories (kcal) purchased on price promotion per week	24.5%	19.8%	18.3%
Mean weekly calories (kcal) purchased on price promotion per person in household	279	156	120
Mean percentage of calories (kcal) purchased on volume promotion per week	13.8%	23.6%	23.9%
Mean weekly calories (kcal) purchased on volume promotion per person in household	198	245	218
Observations	199 886	135 399	118 654
Households	3024	3024	3024

Table 2 provides sociodemographic information for households when they first enter the sample. The KWP survey methods aim to form a representative sample at the UK level. Although our sample includes only households in Scotland, the descriptive statistics fall broadly in line with what would be expected of a representative sample. The mean age of the main shopper is 45 years old, although there is substantial variation in this variable such that the range (18–85 years) captures young adults to the elderly. For comparison, the 2011 Scottish census found a mean age of 40.3 years within the population. As those under 18 are excluded from the sample to be analysed a higher mean age was expected. The mean household size of 2.8 is slightly higher than a mean of around 2.2 found in the 2011 Scottish census. Consistent with a larger household size, more households have children in this sample (40.1%) than found in the census (30.6%). The one variable which may not accurately reflect the Scottish population is household income. It appears that low-income household may be slightly overrepresented in our sample. This issue was also found by Smith *et al* when comparing the UK sample from the KWP with representative data from the Living Cost and Food Survey.^21^

**Table 2:** Descriptive statistics for sample households at first observation (N=3024)

Variable	Sample mean
Age of main shopper	45.0 (13.8) (18–85)
Age of main shopper^2^/100	22.1 (13.3) (3.2–72.3)
Household size	2.8 (1.3) (1–9)
One or more children in household	40.1%
Low annual household income <£20 000	38.3%
Middle annual household income >£19 999 and <£40 000	40.0%
High annual household income >£39 999	21.7%

SD in parentheses and range in brackets for continuous variables only.

Table 3 reports the responsiveness of household purchasing to price, any price promotion and any volume promotion for each product category at multiple points of the purchase volume distribution. The results in table 3 come from multiple regression estimates which are transformed to be on a comparable scale. Full untransformed regression results are available within the online supplemental appendix. For each price or promotion incentive, the estimated response at the mean, median, 25th percentile and 75th percentile of the purchasing volume distribution is provided, expressed in terms of the percentage change in calories (kcal) purchased. In this respect, table 3 provides estimates for the response of average, low-volume and high-volume purchasers. The patterns observed between the 25th and 75th percentiles extend to higher or lower percentiles of the household purchasing distribution.

**Table 3:** Estimated percentage change in calories (kcal) purchased due to incentives across the household purchasing distribution

Dependent variable: calories (kcal) purchased	Biscuits (% change)	Crisps (% change)	Savoury snacks (% change)	χ^2^-test of equality
10% Price reduction—mean	4.3*** (4.2 to 4.4)	8.8*** (8.5 to 9.1)	6.4*** (6.2 to 6.6)	979.4***
10% Price reduction—25th percentile	4.6*** (4.5 to 4.7)	9.1*** (8.8 to 9.5)	7.1*** (6.9 to 7.3)	–
10% Price reduction—median	4.3*** (4.2 to 4.4)	8.8*** (8.5 to 9.2)	6.4*** (6.2 to 6.6)	–
10% Price reduction—75th percentile	4.1*** (4.0 to 4.2)	8.5*** (8.2 to 8.8)	5.7*** (5.5 to 5.9)	–
Any price promo.—mean	37.0*** (35.8 to 38.2)	7.5*** (6.1 to 8.9)	26.4*** (24.9 to 27.9)	950.0***
Any price promo.—25th percentile	35.6*** (34.3 to 37.0)	5.7*** (4.1 to 7.4)	24.2*** (22.5 to 25.9)	–
Any price promo.—median	37.0*** (35.8 to 38.2)	7.5*** (6.0 to 8.9)	26.4*** (24.6 to 28.1)	–
Any price promo.—75th percentile	38.4*** (37.3 to 39.5)	9.3*** (8.0 to 10.6)	28.7*** (27.5 to 29.9)	–
Any volume promo.—mean	72.9*** (71.3 to 74.6)	52.3*** (50.2 to 54.4)	77.8*** (75.7 to 79.8)	380.6***
Any volume promo.—25th percentile	87.1*** (85.2 to 89.1)	58.1*** (55.3 to 61.0)	88.5*** (85.9 to 91.0)	–
Any volume promo.—median	72.8*** (71.3 to 74.3)	52.3*** (50.3 to 54.3)	77.9*** (76.0 to 79.8)	–
Any volume promo.—75th percentile	59.8*** (58.5 to 61.2)	46.6*** (44.6 to 48.7)	67.6*** (66.0 to 69.2)	–
Observations	199 886	135 399	118 654	–
Households	3024	3024	3024	–

95% CI in brackets **p < 0.05, ***p < 0.01.

Price variable refers to a 10% price change (coefficient for a 1% change available in the online supplemental appendix).

Price promotion variable refers to all price promotions pooled.

Volume promotion variable refers to all volume promotions pooled.

Number of observations within categories varies based on the number of purchases per household.

All models include age of main shopper, age^2^ of main shopper, household size, time in panel (years), time since last purchase (weeks), and year dummies (full result available in online supplemental appendix).

Between product categories, table 3 indicates that the scale of the purchasing response to incentives varies significantly. The own-price elasticity of demand is highest for crisps, where a 10% reduction in the price per gram is predicted to increase the mean calories (kcal) purchased by 8.8%. Although mean demand is inelastic (percentage change in demand is less than the percentage change in price) for all product categories, savoury snacks (6.4%) and biscuits (4.3%) are less responsive to price changes than crisps. A χ^2^ test strongly rejects the null hypothesis that at the mean all product categories are equally responsive to price changes (p=2.14e−213). Equivalent null hypotheses are also rejected for the effects of price promotions (p=5.02e−207) and volume promotions (p=2.26e−83). However, the pattern of responsiveness between products varies. Whereas crisps is the product category most responsive to price, it is the least responsive category for both price promotions and volume promotions. Biscuits is the most responsive category to price promotions (37.0%), and savoury snacks is the most responsive category to volume promotions (77.8%).

Within product categories, the mean calories (kcal) purchased by households is more responsive to volume promotions than to price or to price promotions for all product categories. For example, volume promotions increase the calories (kcal) purchased from biscuits by 72.9% compared with 37.0% for price promotions. In all but one case, the responsiveness of demand to any form of promotion is greater than the response to a 10% price reduction. One exception to this may be the crisps category, where price promotions have an approximately comparable effect to a 10% price reduction.

In all cases, the effect estimates at the median are approximately equal to those at the mean, which is expected since the large volume of observations will reduce the influence of any outliers affecting measures of central tendency. Across the household purchasing distributions for each product category, a consistent pattern is observed. As the volume of purchasing increases, households are less responsive to price, less responsive to volume promotions and more responsive to price promotions. However, due to the substantial differences in calories (kcal) purchased between percentiles of the household purchasing distribution, the level change in calories (kcal) purchased could still be greater at higher percentiles despite being less responsive to a specific incentive. For example, combining descriptive statistics from table 1 and regression results from table 3, at the 25th percentile for biscuits, a volume promotion would be expected to increase calories (kcal) purchased by 429 (87.1% of 493). The equivalent purchasing change at the 75th percentile would be 935 kcal (59.8% of 1563). Although such a calculation is not exact, since table 1 includes calories (kcal) purchased on promotion, this example illustrates that the response in calories (kcal) can be larger at the 75th percentile despite a lower responsiveness in percentage terms compared with the 25th percentile.

All analyses reported in table 3 were repeated using a sample that included households with missing income data, and again using a sample without the restriction that households made at least one purchase in each product category. For all product categories, this resulted in only small changes to coefficient estimates that did not change the pattern of results. As such, the findings are robust to our sample selection. The analyses were also repeated with month dummies included to control for a wider range of time effects. This made only small changes to some coefficients and did not alter the interpretation of the findings. The results of all sensitivity analyses are available on request from the authors.

Table 4 provides estimates of demand responsiveness to price and promotions by household income subgroups. Statistically significant differences are observed between income groups in the response of households to price and promotion incentives within the biscuits category only. Therefore, for crisps and savoury snacks, we fail to reject that within product category the effects of specific incentives are homogenous across households of varying purchasing power. For biscuits, responsiveness to volume promotions and price reductions increases with income. For price promotions on biscuits, households in the middle-income category (£20 000–39 999) are most responsive to this incentive.

**Table 4:** Estimated percentage change in calories (kcal) purchased due to incentives across the household income distribution

	Low income	Middle income	High income	χ^2^-test of equality
Product category: biscuits				
10% Price reduction	4.2*** (4.0 to 4.3)	4.4*** (4.2 to 4.5)	4.5*** (4.3 to 4.8)	6.623**
Any price promo.	35.4*** (33.4 to 37.4)	39.1*** (37.2 to 41.0)	36.1*** (33.4 to 38.8)	7.678**
Any volume promo.	70.7*** (67.8 to 73.5)	73.7*** (71.2 to 76.1)	75.2*** (72.1 to 78.4)	4.749*
Observations	78 009	79 432	42 441	
Households	1226	1351	754	
Product category: crisps				
10% Price reduction	8.7*** (8.3 to 9.2)	8.7*** (8.3 to 9.2)	8.9*** (8.4 to 9.5)	0.400
Any price promo.	8.3*** (6.0 to 10.6)	6.9*** (4.7 to 9.0)	7.8*** (5.2 to 10.4)	0.829
Any volume promo.	50.4*** (46.7 to 54.2)	52.9*** (49.9 to 55.9)	53.8*** (49.9 to 57.8)	1.656
Observations	48 281	55 091	32 014	
Households	1211	1338	751	
Product category: savoury snacks				
10% Price reduction	6.2*** (5.9 to 6.6)	6.5*** (6.2 to 6.9)	6.4*** (6.0 to 6.7)	1.889
Any price promo.	25.2*** (22.8 to 27.6)	27.3*** (24.9 to 29.7)	26.6*** (23.8 to 29.5)	1.474
Any volume promo.	76.2*** (72.9 to 79.5)	77.5*** (74.2 to 80.8)	79.8*** (76.0 to 83.6)	1.941
Observations	40 190	47 868	30 578	
Households	1212	1342	745	

95% CI in brackets *p < 0.10, **p < 0.05, ***p < 0.01.

Number of observations within categories varies based on the number of purchases per household.

All models include age of main shopper, age^2^ of main shopper, household size, time in panel (years), time since last purchase (weeks), and year dummies (full results available in online supplemental appendix).

Between households with and without children, variation in the response of calories (kcal) purchased to price and promotions is observed within each product category for at least one form of incentive, as detailed in table 5. For crisps, the demand of households with children are more responsive to price than households without children (p=3.43e–7). For savoury snacks, the demand of households with children are more responsive to price promotions (p=1.21e–6) and volume promotions (p=0.099) than households without children. Within these two product categories, no other statistically significant differences are observed for demand responsiveness to other incentives. For biscuits only, the calories (kcal) purchased by households with children are more responsive than households without children to all specified forms of incentives (price p=0.001, price promotion p=6.92e–9, and volume promotion p=1.17e–5). Across product categories, in all cases where statistically significant differences are observed, households with children are more responsive to incentives than households without children.

**Table 5:** Estimated percentage change in calories (kcal) purchased due to incentives by household type

	With children	No children	χ^2^-test of equality
Product category: biscuits			
10% Price reduction	4.6*** (4.4 to 4.7)	4.2*** (4.1 to 4.3)	11.72***
Any price promo.	41.5*** (39.5 to 43.4)	34.3*** (32.7 to 35.8)	33.56***
Any volume promo.	77.4*** (74.7 to 80.1)	69.8*** (67.8 to 71.9)	19.22***
Observations	72 843	127 041	
Households	1261	1896	
Product category: crisps			
10% Price reduction	9.7*** (9.3 to 10.2)	8.3*** (7.9 to 8.6)	25.99***
Any price promo.	6.5*** (4.3 to 8.8)	8.3*** (6.6 to 10.1)	1.580
Any volume promo.	52.1*** (48.9 to 55.4)	52.4*** (49.8 to 55.1)	0.019
Observations	50 036	85 358	
Households	1259	1894	
Product category: savoury snacks			
10% Price reduction	6.5*** (6.2 to 6.7)	6.3*** (6.1 to 6.6)	0.433
Any price promo.	30.2*** (28.0 to 32.3)	22.8*** (20.8 to 24.9)	23.55***
Any volume promo.	79.4*** (76.5 to 82.3)	76.0*** (73.3 to 78.8)	2.724*
Observations	56 008	62 640	
Households	1258	1896	

95% CI in brackets *p < 0.10, **p < 0.05, ***p < 0.01.

Number of observations within categories varies based on the number of purchases per household.

All models include age of main shopper, age^2^ of main shopper, household size, time in panel (years), time since last purchase (weeks), and year dummies (full results available in online supplemental appendix).

## Discussion

The analysis in this paper uses observed changes in consumer demand for HFSS foods resulting from in-store incentives as an indicator of responsiveness to potential policy instruments. The instruments implied by the comparison in table 3 are a 10% tax on the price per gram of a product, a total ban on all price promotions, and a total ban on all volume promotions. We find clear evidence that promotions cause households to purchase more than would be expected in the absence of promotions. Therefore, policy instruments targeting promotions are predicted to be effective in reducing the calories from HFSS foods purchased by households.

When interpreting the results, it is assumed that households respond symmetrically to price and promotion incentives and disincentives. That is, the removal of a promotion would decrease consumption by the same amount as consumption increases when a promotion is introduced. Although this is a standard assumption in demand analysis, there is some evidence that households may respond asymmetrically to price changes for foods.^22–24^ In the context of HFSS foods, this asymmetry became less evident as individual products were aggregated into product groups, and the inclusion of household fixed effects also often reduced the extent of asymmetries.^22^ Both of these factors apply to the analysis in this paper and provide some empirical foundation for the simplifying assumption of symmetric response by households. Furthermore, studies investigating demand asymmetry have not considered separately the source of price changes and how these might interact with the separate behavioural effects of promotions. However, if a policy change was to be introduced, it is likely that suppliers and consumers would alter their behaviour to counteract the policy and this could contribute to asymmetric response. Therefore, while it is unlikely that demand asymmetry would substantially alter our conclusions, the results reported above predict the likely pattern of changes in household response, rather than precise scale of changes.

Although heterogeneity in instrument effectiveness is observed in the results, it is also the case that a total ban of volume promotions is clearly indicated as the most effective instrument to reduce calories purchased by households, assuming retailers do not undermine the ban by increasing price promotions. Even with a switch to price promotions, some effect could be achieved; a ban on volume promotions for alcohol in Scotland showed significant reductions for some alcohol products (wine and ready to drink), and a significant overall reduction despite widely reported price reductions being offered.^25^ For all product categories analysed, a total ban on volume promotions is predicted to reduce household purchasing of calories (kcal) from HFSS foods by at least 50%. An effect of this size may seem large but appears plausible when considered in the context of common volume promotions. For example, ‘buy one, get one free’ doubles the volume purchased by a household at a given price. Such promotions require households to purchase a minimum volume, which would not be the case for a price promotion with an equivalent price per gram offered to consumers.

The results in this paper are consistent with those reported by Public Health England in important ways.^14^ In particular, promotions are shown to increase purchasing by households, and volume promotions increase purchases to a greater extent than price promotion. Direct comparison of point estimates cannot be made due to substantial differences in the methods employed to form estimates,^14^ particularly in terms of measuring volume and controlling for unobserved heterogeneity. Despite this, the same overarching conclusions would be made regardless of the approach employed. Although the magnitude of the effects is substantially smaller in Public Health England’s analysis,^14^ there are a number of potential reasons for this. Public Health England decompose the volume increases observed from promotions and find that across all product categories 18% of volume increases would not have occurred if not for a promotion.^14^ For volume promotions with a discount of between 45% and 55%, the estimate of increased purchasing is 27% for all HFSS and non-HFSS foods combined. Estimates are not available that simultaneously disaggregate at both the product and promotion type. Disaggregation at only the product category level indicates that the incremental increases in purchase volume due to promotions are greater for HFSS foods, which are also shown to have higher levels of discount.^14^ Therefore, based on the Public Health England estimates,^14^ it would be expected that increased purchasing from volume discounts on HFSS foods would be above 27%.

Our results suggest that volume promotions, effectively price per gram offers that are conditional on a minimum volume of purchase, lead to substantial increases in calories (kcal) purchased by households. Therefore, if a single policy instrument was to be used across the product categories analysed, a volume promotions ban is predicted to be most effective. A ban on price promotions cannot achieve the same levels of reductions in calories (kcal) demanded, whereas a substantial tax on the price per gram (65% or more) is predicted to be required to achieve reductions equivalent to a ban on volume promotions, plus a tax may be more difficult to administer relative to any form of promotion ban.

A further benefit of a ban on volume promotions as a policy instrument is that reductions in purchasing would not be most severe within households with relatively low income. This is consistent with the Public Health England findings that lower income shoppers are less likely to make purchases on promotions,^14^ and with Food Standards Scotland finding of little difference in the percentage of calories purchased on promotion by area deprivation.^9^ For crisps and savoury snacks, table 4 indicates that all purchase incentives affect income groups equally. For biscuits only, there is some evidence of responsiveness increasing with income. This creates the possibility of greater health benefits among higher income groups from legislation limiting incentives for HFSS foods. However, this effect is most uncertain for volume promotions where the difference in response between income groups is only statistically significant at the 10% level. Other incentives for biscuits, price reductions and price promotions, indicate differences between income groups at a higher level of statistical significance. By targeting volume promotions, policy-makers can limit the risk of unintentionally widening income-related health inequalities through the introduction of a policy.

The results in table 5 indicate that households with children are relatively more responsive to incentives, in some cases. This would be a desirable outcome where the objective of a policy is to reduce childhood obesity. Public Health England found that families were more likely to purchase based on promotions in 2013–2015 but not in 2017–2018.^14^ Public Health England attributes the absence of this pattern in later data to the increased use by families of discounter outlets, which rarely use promotions.^14^ Therefore, this element of our results must be treated with some caution in the absence of further research.

One potentially less desirable feature of a ban on volume promotions is that high-volume consumers appear relatively less responsive to this form of policy intervention. Therefore, such a policy may be criticised for not targeting problematic consumers who habitually purchase substantial volumes of HFSS products. However, as illustrated in the results section, a lower percentage response combined with a higher level of calories purchased would be expected to result in a more substantial calorie reduction overall. Therefore, such a criticism would appear to be unfounded.

It should be noted that any demand response to promotion-based incentives will be a combination of a price per gram effect and the effect of a product being promoted. By standardising prices across many products, such that they are expressed as price per gram of the product, our analysis was able to identify distinct price and promotion effects. However, this does lead to the effect of promotion instruments being understated in our analysis, since the promotion will also influence either the price paid, or the weight bought. Importantly, this is true for both price and volume promotions, which enable valid comparison to be made across potential policy instruments. Sensitivity analysis (results available on request) was conducted with price excluded from the model specification, such that the promotion variables captured the combined price and promotion effect. In all cases, volume promotions were shown to have at least a 45% larger effect on calories (kcal) purchased than price promotions which supports the earlier conclusion that restricting volume promotions would be the more effective policy instrument to reduce calories of HFSS foods purchased by households. In future work it would be desirable to disaggregate further to specific price or volume promotions since the response of households may vary within these categories of promotions. Such disaggregation may also indicate patterns of promotions that result in high purchasing by households.

Within this study a large volume of consumer data with accurate product-level information was used. This enabled reliable analysis at product category levels with greater disaggregation than most of the extant literature. However, some level of under-reporting is likely within the KWP and purchases of HFSS for consumption outside the home are excluded. The model specification attempts to limit this source of potential bias by controlling for time-invariant household characteristics and the time a household has spent in the panel.

Another feature of this study is that the data covers a period (2006–2012) from prior to the global financial crisis to after the crisis. Public Health England indicated that the level of promotions increased through the period to 2010 and then stabilised.^14^ Therefore, it is likely that the data used in this study remain representative of the current retail environment. Analysis of UK survey data indicated that households’ main response to food prices increasing in real terms due to the crisis was to increase expenditure, although there was also a reduction in quantity purchased and households ‘traded down’ by substituting cheaper versions of similar products. This response was seen in both the short term (2007–2010) and longer term (2007–2015).^26 27^ While the share of expenditure going to food and non-alcoholic drink did rise sharply in 2008 (from 15.2% to 16.8%), it remained above pre-the 2008 level beyond 2012, suggesting that household budgets also adjusted in the longer term. Additional sensitivity analysis was conducted that split the sample period into a precrisis period (2006–2007), crisis period (2008–2010), and postcrisis period (2011–2012). Although this results in some changes to the coefficient estimates, the main finding that volume promotions substantially increase purchases across the three product groups to a far greater extent than other incentives remained apparent (results available on request). Therefore, there is no evidence that the effects of the financial crisis altered the findings of this analysis.

The model specification used in this study is relatively simple in the sense that only own-price and own-promotion effects are estimated. A more complex model could look to account for substitution across product categories, as in Smith *et al*.^21^ However, such models would require substantial further assumptions regarding the relationship between products on specific promotions which would be highly speculative a priori. By contrast, the relatively simple model specification adopted here enabled the separate identification of price and promotion effects, with the clear implications that policy instruments focused on restricting volume promotions are likely to be most effective in reducing calories consumed from HFSS. Reassuringly, our estimates of own-price elasticities are broadly comparable with those in Smith *et al* using similar data but a different model specification.^21^ In future research, it would be of interest to first extend the analysis to a wider range of product categories, with confectionery being one category that could be particularly informative. After including a greater number of product categories, accounting for interactions between the demand for products could provide further insights.

## Conclusion

Incentivising consumers to purchase fewer HFSS products is an aim for many governments. The analysis in this paper has demonstrated that the sensitivity of consumer demand to specific price and promotion incentives varies between product categories. Although the magnitude of the changes in demand vary, for all product categories analysed (biscuits, crisps and savoury snacks) consumers are most responsive to volume promotions. Therefore, the most effective policy instrument to reduce the calorie intake from these products may be a ban on volume promotions. However, it should be noted that the effect of a ban only on volume promotions could be weakened by retailers increasing the use of price promotions. It is encouraging that a ban on volume promotions is a central component of policy being proposed by the UK Government to be introduced in England from 2022. Similar legislation for Scotland is being considered. The analysis presented here provides further evidence that a ban on volume promotions could have substantial benefits to population health.

## Data Availability

Data may be obtained from a third party and are not publicly available. Kantar Worldpanel data are not publicly available but can be purchased from Kantar Worldpanel (http://www.kantarworldpanel.com). The authors are not legally permitted to share the data used for this study.
