# Supplementary material for: Longitudinal study of the effects of price and promotion incentives on purchases of unhealthy foods: evidence for restricting food promotions
Source: BMJ Nutr Prev Health. 2022 Mar 4;5(1):62–71. doi: 10.1136/bmjnph-2021-000323 (PMC9237875; doi:10.1136/bmjnph-2021-000323)
Supplement: Supplementary data [file bmjnph-2021-000323supp001.pdf]

Appendix

Table A1. Estimated Change in Log Calories (kcal) of Biscuits Purchased Due to Incentives Across the Household Purchasing Distribution.

Dependent variable: Log of calories (kcal) purchase	Biscuits Mean	Biscuits Median	Biscuits 25th percentile	Biscuits 75th Percentile
Log of real price (£) per gram	-0.432***	-0.432***	-0.456***	-0.408***
	(0.006)	(0.005)	(0.005)	(0.006)
Price promotion in week	0.315***	0.315***	0.305***	0.325***
	(0.005)	(0.004)	(0.005)	(0.004)
Volume promotion in week	0.548***	0.547***	0.627***	0.469***
	(0.005)	(0.004)	(0.005)	(0.004)
Shopper age	0.012	0.012	0.012	0.0130
	(0.008)	(0.008)	(0.008)	(0.009)
Shopper age squared/100	-0.011*	-0.011*	-0.011*	-0.011
	(0.006)	(0.007)	(0.006)	(0.007)
Household size	0.043***	0.043***	0.035***	0.050***
	(0.010)	(0.011)	(0.012)	(0.012)
Years in panel	0.021***	0.021***	0.027***	0.015**
	(0.005)	(0.005)	(0.007)	(0.007)
Weeks since last purchase	-0.001***	-0.001***	-0.002***	-0.002***
	(0.000)	(0.000)	(0.000)	(0.000)
Year (base 2006): 2007	-0.047***	-0.047***	-0.053***	-0.042***
	(0.011)	(0.012)	(0.014)	(0.013)
Year (base 2006): 2008	-0.053***	-0.053***	-0.065***	-0.041**
	(0.017)	(0.017)	(0.022)	(0.020)
Year (base 2006): 2009	-0.069***	-0.069***	-0.076***	-0.062**
	(0.024)	(0.024)	(0.029)	(0.028)
Year (base 2006): 2010	-0.114***	-0.114***	-0.128***	-0.101***
	(0.032)	(0.031)	(0.038)	(0.037)
Year (base 2006): 2011	-0.158***	-0.158***	-0.176***	-0.140***
	(0.039)	(0.041)	(0.045)	(0.047)
Year (base 2006): 2012	-0.180***	-0.180***	-0.206***	-0.154***
	(0.047)	(0.049)	(0.054)	(0.055)
Observations	199886	199886	199886	199886
Households	3024	3024	3024	3024
R^2	0.248	-	-	-

Clustered standard errors in parentheses (bootstrapped except for estimates at the mean)
* p < 0.10, ** p < 0.05, *** p < 0.01

Table A2. Estimated Change in Log Calories (kcal) of Crisps Purchased Due to Incentives Across the Household Purchasing Distribution.

Dependent variable: Log of calories (kcal) purchase	Crisps Mean	Crisps Median	Crisps 25th percentile	Crisps 75th Percentile
Log of real price (£) per gram	-0.882***	-0.882***	-0.911***	-0.852***
	(0.014)	(0.018)	(0.018)	(0.015)
Price promotion in week	0.072***	0.072***	0.056***	0.089***
	(0.007)	(0.007)	(0.008)	(0.006)
Volume promotion in week	0.421***	0.421***	0.458***	0.383***
	(0.007)	(0.007)	(0.009)	(0.007)
Shopper age	-0.011	-0.011	-0.010	-0.012
	(0.009)	(0.010)	(0.012)	(0.009)
Shopper age squared/100	0.003	0.003	-0.002	0.009
	(0.008)	(0.010)	(0.009)	(0.009)
Household size	0.033***	0.033**	0.032**	0.034**
	(0.013)	(0.013)	(0.015)	(0.014)
Years in panel	0.007	0.007	0.015**	-0.002
	(0.006)	(0.006)	(0.006)	(0.006)
Weeks since last purchase	-0.002***	-0.002***	-0.002***	-0.001***
	(0.000)	(0.000)	(0.000)	(0.000)
Year (base 2006): 2007	0.025**	0.025**	0.033***	0.017
	(0.012)	(0.012)	(0.013)	(0.012)
Year (base 2006): 2008	0.081***	0.081***	0.093***	0.069***
	(0.018)	(0.019)	(0.023)	(0.018)
Year (base 2006): 2009	0.082***	0.082***	0.087***	0.076***
	(0.025)	(0.026)	(0.033)	(0.023)
Year (base 2006): 2010	0.075**	0.075**	0.081*	0.069**
	(0.032)	(0.034)	(0.044)	(0.029)
Year (base 2006): 2011	0.067*	0.067	0.072	0.061*
	(0.040)	(0.042)	(0.052)	(0.036)
Year (base 2006): 2012	0.060	0.060	0.063	0.057
	(0.047)	(0.051)	(0.063)	(0.043)
Observations	135399	135399	135399	135399
Households	3024	3024	3024	3024
R^2	0.302	-	-	-

Clustered standard errors in parentheses (bootstrapped except for estimates at the mean)
* p < 0.10, ** p < 0.05, *** p < 0.01

Table A3. Estimated Change in Log Calories (kcal) of Snacks Purchased Due to Incentives Across the Household Purchasing Distribution.

Dependent variable: Log of calories (kcal) purchase	Snacks Mean	Snacks Median	Snacks 25th percentile	Snacks 75th Percentile
Log of real price (£) per gram	-0.638***	-0.639***	-0.708***	-0.569***
	(0.010)	(0.010)	(0.011)	(0.011)
Price promotion in week	0.234***	0.234***	0.217***	0.252***
	(0.006)	(0.007)	(0.007)	(0.005)
Volume promotion in week	0.575***	0.576***	0.634***	0.517***
	(0.006)	(0.005)	(0.007)	(0.005)
Shopper age	-0.008	-0.008	-0.011	-0.006
	(0.009)	(0.012)	(0.013)	(0.009)
Shopper age squared/100	0.001	0.001	0.005	-0.003
	(0.008)	(0.008)	(0.010)	(0.008)
Household size	0.040***	0.040***	0.038***	0.042***
	(0.011)	(0.011)	(0.014)	(0.011)
Years in panel	0.013**	0.013**	0.025***	0.001
	(0.006)	(0.006)	(0.007)	(0.006)
Weeks since last purchase	-0.001***	-0.001***	-0.001***	-0.001**
	(0.000)	(0.000)	(0.000)	(0.000)
Year (base 2006): 2007	0.000	-0.000	-0.007	0.001
	(0.013)	(0.013)	(0.015)	(0.014)
Year (base 2006): 2008	0.032	0.032	0.016	0.048**
	(0.021)	(0.024)	(0.024)	(0.023)
Year (base 2006): 2009	0.028	0.028	0.011	0.045
	(0.029)	(0.032)	(0.035)	(0.029)
Year (base 2006): 2010	-0.002	-0.002	-0.022	0.019
	(0.037)	(0.043)	(0.046)	(0.039)
Year (base 2006): 2011	-0.012	-0.012	-0.049	0.026
	(0.047)	(0.054)	(0.056)	(0.047)
Year (base 2006): 2012	-0.003	-0.004	-0.053	0.047
	(0.056)	(0.065)	(0.068)	(0.056)
Observations	118654	118654	118654	118654
Households	3024	3024	3024	3024
R^2	0.330	-	-	-

Clustered standard errors in parentheses (bootstrapped except for estimates at the mean)
* p < 0.10, ** p < 0.05, *** p < 0.01

Table A4. Estimated Change in Log Calories (kcal) of Biscuits Purchased Due to Incentives Across the Household Income Distribution.

Dependent variable: Log of calories (kcal) purchase	Biscuits Low income	Biscuits Middle income	Biscuits High income
Log of real price (£) per gram	-0.418***	-0.436***	-0.453***
	(0.008)	(0.009)	(0.011)
Price promotion in week	0.303***	0.330***	0.308***
	(0.007)	(0.007)	(0.010)
Volume promotion in week	0.534***	0.552***	0.561***
	(0.009)	(0.007)	(0.009)
Shopper age	-0.005	0.035	-0.001
	(0.015)	(0.032)	(0.016)
Shopper age squared/100	0.004	-0.022**	-0.005
	(0.011)	(0.011)	(0.017)
Household size	0.060***	0.044**	-0.008
	(0.021)	(0.020)	(0.020)
Years in panel	0.019**	0.022**	0.024**
	(0.008)	(0.008)	(0.011)
Weeks since last purchase	-0.002***	-0.001	-0.001*
	(0.001)	(0.001)	(0.001)
Year (base 2006): 2007	-0.055***	-0.042	-0.041*
	(0.018)	(0.033)	(0.021)
Year (base 2006): 2008	-0.061**	-0.044	-0.032
	(0.028)	(0.059)	(0.032)
Year (base 2006): 2009	-0.086**	-0.071	-0.029
	(0.041)	(0.090)	(0.042)
Year (base 2006): 2010	-0.135**	-0.120	-0.077
	(0.054)	(0.120)	(0.054)
Year (base 2006): 2011	-0.180***	-0.180	-0.102
	(0.068)	(0.150)	(0.065)
Year (base 2006): 2012	-0.194**	-0.212	-0.135*
	(0.082)	(0.180)	(0.076)
Observations	78009	79432	42441
Households	1226	1351	754
R^2	0.234	0.252	0.262

Clustered standard errors in parentheses
* $p < 0.10$, ** $p < 0.05$, *** $p < 0.01$

Kopasker D, *et al. bmjnph* 2022; 5:e000323. doi: 10.1136/bmjnph-2021-000323

Table A5. Estimated Change in Log Calories (kcal) of Crisps Purchased Due to Incentives Across the Household Income Distribution.

Dependent variable: Log of calories (kcal) purchase	Crisps Low income	Crisps Middle income	Crisps High income
Log of real price (£) per gram	-0.873***	-0.874***	-0.894***
	(0.024)	(0.022)	(0.028)
Price promotion in week	0.080***	0.067***	0.075***
	(0.011)	(0.010)	(0.013)
Volume promotion in week	0.408***	0.424***	0.431***
	(0.013)	(0.010)	(0.013)
Shopper age	-0.031	-0.027	0.032*
	(0.021)	(0.022)	(0.017)
Shopper age squared/100	0.026*	0.003	-0.040**
	(0.015)	(0.013)	(0.017)
Household size	-0.013	0.010	0.080***
	(0.031)	(0.024)	(0.024)
Years in panel	0.020**	0.004	-0.006
	(0.010)	(0.009)	(0.011)
Weeks since last purchase	-0.001***	-0.001***	-0.002***
	(0.000)	(0.000)	(0.001)
Year (base 2006): 2007	0.007	0.034	0.062***
	(0.030)	(0.024)	(0.021)
Year (base 2006): 2008	0.060	0.095**	0.095***
	(0.048)	(0.039)	(0.029)
Year (base 2006): 2009	0.037	0.119**	0.107***
	(0.072)	(0.057)	(0.039)
Year (base 2006): 2010	0.032	0.114	0.110**
	(0.096)	(0.075)	(0.049)
Year (base 2006): 2011	0.005	0.130	0.101*
	(0.120)	(0.094)	(0.059)
Year (base 2006): 2012	-0.025	0.142	0.112
	(0.144)	(0.113)	(0.070)
Observations	48281	55091	32014
Households	1211	1338	751
R^2	0.296	0.300	0.313

Clustered standard errors in parentheses
* $p < 0.10$, ** $p < 0.05$, *** $p < 0.01$

Table A6. Estimated Change in Log Calories (kcal) of Snacks Purchased Due to Incentives Across the Household Income Distribution.

Dependent variable: Log of calories (kcal) purchase	Snacks Low income	Snacks Middle income	Snacks High income
Log of real price (£) per gram	-0.623***	-0.654***	-0.636***
	(0.017)	(0.016)	(0.018)
Price promotion in week	0.225***	0.241***	0.236***
	(0.010)	(0.010)	(0.011)
Volume promotion in week	0.566***	0.574***	0.586***
	(0.010)	(0.009)	(0.011)
Shopper age	-0.006	0.069***	0.017
	(0.017)	(0.025)	(0.014)
Shopper age squared/100	0.011	0.002	-0.043***
	(0.013)	(0.014)	(0.015)
Household size	0.072***	0.050**	-0.007
	(0.026)	(0.020)	(0.025)
Years in panel	0.008	0.018*	0.011
	(0.011)	(0.010)	(0.012)
Weeks since last purchase	-0.001**	-0.001**	-0.002***
	(0.000)	(0.000)	(0.001)
Year (base 2006): 2007	-0.026	-0.072**	0.041*
	(0.024)	(0.028)	(0.022)
Year (base 2006): 2008	0.011	-0.093**	0.073**
	(0.039)	(0.047)	(0.035)
Year (base 2006): 2009	-0.003	-0.191***	0.100**
	(0.055)	(0.068)	(0.045)
Year (base 2006): 2010	-0.041	-0.301***	0.079
	(0.074)	(0.091)	(0.054)
Year (base 2006): 2011	-0.061	-0.396***	0.086
	(0.092)	(0.115)	(0.067)
Year (base 2006): 2012	-0.053	-0.482***	0.117
	(0.111)	(0.139)	(0.079)
Observations	40190	47868	30578
Households	1212	1342	745
R^2	0.320	0.334	0.337

Clustered standard errors in parentheses
* $p < 0.10$, ** $p < 0.05$, *** $p < 0.01$

Kopasker D, *et al. bmjnph* 2022; 5:e000323. doi: 10.1136/bmjnph-2021-000323

Table A7. Estimated Change in Log Calories (kcal) of Biscuits Purchased Due to Incentives by Household Type

Dependent variable: Log of calories (kcal) purchase	Biscuits No children	Biscuits With children
Log of real price (£) per gram	-0.418***	-0.456***
	(0.007)	(0.008)
Price promotion in week	0.295***	0.347***
	(0.006)	(0.007)
Volume promotion in week	0.530***	0.573***
	(0.006)	(0.008)
Shopper age	-0.018	0.040*
	(0.011)	(0.021)
Shopper age squared/100	0.013	-0.031
	(0.008)	(0.023)
Household size	0.035**	0.065***
	(0.015)	(0.019)
Years in panel	0.013**	0.034***
	(0.006)	(0.009)
Weeks since last purchase	-0.001***	-0.002**
	(0.000)	(0.001)
Year (base 2006): 2007	-0.035**	-0.078***
	(0.014)	(0.019)
Year (base 2006): 2008	-0.030	-0.106***
	(0.022)	(0.032)
Year (base 2006): 2009	-0.031	-0.154***
	(0.031)	(0.046)
Year (base 2006): 2010	-0.065	-0.223***
	(0.040)	(0.060)
Year (base 2006): 2011	-0.109**	-0.265***
	(0.050)	(0.074)
Year (base 2006): 2012	-0.135**	-0.285***
	(0.059)	(0.088)
Observations	127041	72843
Households	1896	1261
R^2	0.235	0.268

Clustered standard errors in parentheses
* $p < 0.10$, ** $p < 0.05$, *** $p < 0.01$

Kopasker D, *et al. bmjnph* 2022; 5:e000323. doi: 10.1136/bmjnph-2021-000323

Table A8. Estimated Change in Log Calories (kcal) of Crisps Purchased Due to Incentives by Household Type

Dependent variable: Log of calories (kcal) purchase	Crisps No children	Crisps With children
Log of real price (£) per gram	-0.826***	-0.972***
	(0.018)	(0.023)
Price promotion in week	0.080***	0.063***
	(0.008)	(0.011)
Volume promotion in week	0.421***	0.420***
	(0.009)	(0.011)
Shopper age	-0.036***	0.021
	(0.012)	(0.018)
Shopper age squared/100	0.022**	-0.030
	(0.011)	(0.020)
Household size	0.014	0.0092
	(0.021)	(0.025)
Years in panel	0.006	0.007
	(0.007)	(0.009)
Weeks since last purchase	-0.002***	-0.001**
	(0.000)	(0.001)
Year (base 2006): 2007	0.028*	0.020
	(0.017)	(0.017)
Year (base 2006): 2008	0.083***	0.083***
	(0.026)	(0.028)
Year (base 2006): 2009	0.085**	0.086**
	(0.036)	(0.037)
Year (base 2006): 2010	0.095**	0.049
	(0.047)	(0.047)
Year (base 2006): 2011	0.078	0.063
	(0.057)	(0.057)
Year (base 2006): 2012	0.072	0.056
	(0.067)	(0.067)
Observations	85358	50036
Households	1894	1259
R^2	0.289	0.322

Clustered standard errors in parentheses
* $p < 0.10$, ** $p < 0.05$, *** $p < 0.01$

Kopasker D, et al. bmjnph 2022; 5:e000323. doi: 10.1136/bmjnph-2021-000323

Table A9. Estimated Change in Log Calories (kcal) of Snacks Purchased Due to Incentives by Household Type

Dependent variable:<br>Log of calories (kcal) purchase	Snacks<br>No children	Snacks<br>With children
Log of real price (£) per gram	-0.633***	-0.646***
	(0.014)	(0.014)
Price promotion in week	0.206***	0.264***
	(0.008)	(0.008)
Volume promotion in week	0.565***	0.584***
	(0.008)	(0.008)
Shopper age	-0.028**	0.016
	(0.012)	(0.015)
Shopper age squared/100	0.015	-0.020
	(0.011)	(0.017)
Household size	0.053***	0.025
	(0.017)	(0.023)
Years in panel	0.0001	0.028***
	(0.009)	(0.009)
Weeks since last purchase	-0.001***	-0.001**
	(0.000)	(0.001)
Year (base 2006): 2007	0.030*	-0.028*
	(0.017)	(0.017)
Year (base 2006): 2008	0.083***	-0.012
	(0.028)	(0.028)
Year (base 2006): 2009	0.097***	-0.037
	(0.036)	(0.039)
Year (base 2006): 2010	0.085*	-0.088*
	(0.045)	(0.050)
Year (base 2006): 2011	0.092*	-0.118*
	(0.056)	(0.064)
Year (base 2006): 2012	0.112*	-0.124
	(0.066)	(0.076)
Observations	62640	56008
Households	1896	1258
R^2	0.327	0.334

Clustered standard errors in parentheses

* $p < 0.10$, ** $p < 0.05$, *** $p < 0.01$

Kopasker D, *et al. bmjnph* 2022; 5:e000323. doi: 10.1136/bmjnph-2021-000323
